# Development and internal validation of a machine learning model for predicting acute chemotherapy-induced nausea and vomiting after intra-arterial interventional therapy in patients with liver cancer

**DOI:** 10.1016/j.apjon.2026.101040

**Published:** 2026-10-03

**Authors:** Yan Lu, Ruishuang Zheng, Haipeng Yu, Xueling Yang, Yanan Liu, Yongjian Wang, Dongjun Liu

**Affiliations:** a Department of Interventional Oncology, Tianjin Medical University Cancer Hospital and Institute, National Clinical Research Center for Cancer, Key Laboratory of Cancer Prevention and Therapy, Tianjin's Clinical Research Center for Cancer, Tianjin, China; b Department of Hepatobiliary Cancer, Tianjin Medical University Cancer Hospital and Institute, National Clinical Research Center for Cancer, Key Laboratory of Cancer Prevention and Therapy, Tianjin's Clinical Research Center for Cancer, Tianjin, China; c Tianjin Medical University Cancer Hospital and Institute, National Clinical Research Center for Cancer, National Clinical Research Center for Interventional Medicine, Key Laboratory of Cancer Prevention and Therapy, Tianjin's Clinical Research Center for Cancer, Tianjin, China

**Keywords:** Liver cancer, Interventional therapy, Nausea and vomiting, Prediction model, Machine learning

## Abstract

**Objective:**

To develop and internally validate an interpretable machine learning model for predicting acute chemotherapy-induced nausea and vomiting (ACINV) after intra-arterial interventional therapy (IAIT) in patients with primary liver cancer.

**Methods:**

This retrospective cohort included 1062 patients who underwent IAIT in China. ACINV was defined as Common Terminology Criteria for Adverse Events (CTCAE) grade ≥ 2 nausea or vomiting within 24 hours. Patients were allocated to a training set (*n* = 638) and a held-out internal validation set (*n* = 424). Seven algorithms were compared using nested stratified five-fold cross-validation. Outer folds generated out-of-fold (OOF) predictions, whereas inner folds supported tuning and threshold selection. Imputation, encoding, standardization, and least absolute shrinkage and selection operator (LASSO) selection were fitted only to training observations in each partition. Preprocessing was re-estimated within folds used for LASSO penalty selection. SMOTE was applied only within model-fitting partitions after predictor selection. Algorithms were ranked by pooled OOF area under the curve (AUC), with the Brier score resolving ties. The selected pipeline was refitted on the complete training set and evaluated in internal validation.

**Results:**

ACINV occurred in 380 patients (35.8%). The final refit retained 11 predictors. Random forest achieved the highest pooled OOF AUC (0.941; 95% CI, 0.923–0.958) and a Brier score of 0.092. In internal validation, its AUC was 0.962 (95% CI, 0.946–0.978), Brier score was 0.073, calibration intercept was −0.090, and calibration slope was 1.056. SHAP analysis supported model interpretation, and the locked model was incorporated into a prototype calculator.

**Conclusions:**

The model showed promising internal performance. External validation and prospective impact evaluation are required before clinical implementation.

**Trial registration:**

This study was registered in Chinese Clinical Trial Registry (ChiCTR-2500110585).

## Introduction

Among patients with liver cancer, acute chemotherapy-induced nausea and vomiting following intra-arterial interventional therapy (ACINV-IAIT) remains one of the most common and distressing adverse events, largely attributable to the high local concentrations of chemotherapeutic agents delivered directly into the tumor-feeding arteries.^1^ Severe nausea and vomiting may result in aspiration, electrolyte imbalances, malnutrition, and even treatment interruption or delay, ultimately prolonging hospitalizations.^2^ These complications not only substantially impair patients’ physical and psychological well-being but also impose a considerable economic burden on health care systems.

Importantly, patients with liver cancer constitute a uniquely vulnerable population. Many patients commonly exhibit portal hypertension and concomitant esophagogastric varices.^3^ Recurrent episodes of ACINV may increase intra-abdominal pressure and precipitate the rupture of fragile variceal vessels, thereby elevating the risk of gastrointestinal bleeding and potentially leading to mortality.

Although prophylactic antiemetic agents are widely used and remain essential for the prevention of ACINV, a uniform antiemetic strategy may be suboptimal. Patients at higher risk may receive insufficient prophylaxis, whereas those at lower risk may be exposed to unnecessary medication burden, treatment-related adverse effects, and costs.^4^ Furthermore, liver dysfunction may alter drug metabolism and increase susceptibility to treatment-related toxicities, making individualized supportive care particularly important in this population. Therefore, timely identification of patients at high risk for ACINV-IAIT is essential to support more appropriate and individualized prophylactic strategies, enable oncology nurses to implement targeted preventive care and early symptom warning, and ultimately reduce symptom burden and improve patients’ quality of life.

Based on the route of administration, intra-arterial interventional therapies can be categorized into transarterial chemoembolization (TACE) and hepatic arterial infusion chemotherapy (HAIC).^5^ TACE and HAIC are two intra-arterial treatment modalities used in the management of liver cancer. TACE involves the transarterial delivery of chemotherapeutic agents combined with embolization, whereas HAIC involves continuous intra-arterial infusion of chemotherapy.^6^, ^7^, ^8^ Compared with conventional intravenous chemotherapy, intra-arterial interventional therapy exhibits distinct characteristics in terms of the incidence, onset pattern, and predictors of ACINV, largely owing to differences in drug dosage, administration routes, and pharmacological mechanisms.^9^^,^^10^ To facilitate the prediction and prevention of ACINV, several studies have developed risk prediction models for ACINV in patients receiving intravenous chemotherapy across different cancer populations.^11^, ^12^, ^13^ However, these models are limited by conventional statistical approaches, which often demonstrate limited predictive accuracy, methodological deficiencies, and inadequate interpretability, thereby restricting their applicability in clinical decision-making.^14^ To date, no dedicated prediction model has been established to systematically integrate clinical variables and quantitatively assess the risk of ACINV-IAIT in patients with liver cancer.

Recently, machine learning algorithms have gained increasing attention for their potential applications in disease prediction and early diagnosis.^15^ Owing to their powerful data-learning capabilities, these algorithms can effectively capture complex interactions and nonlinear relationships among variables, thereby offering new opportunities for improving the prediction of ACINV-IAIT. However, despite their superior predictive performance, many machine learning models remain difficult to interpret owing to their inherent complexity, which limits their translation into routine clinical practice.^16^ Consequently, achieving an optimal balance between predictive accuracy and clinical interpretability has become a critical consideration in the development of machine learning-based clinical prediction tools.

Therefore, this study aimed to develop and internally validate a prediction model for ACINV after IAIT in patients with primary liver cancer. We compared seven candidate machine learning algorithms using a leakage-controlled nested cross-validation framework, selected a final model using out-of-fold performance in the training set, and evaluated the final model once in a held-out internal-validation set. We additionally used Shapley additive explanations (SHAP) to describe global and individual-level model predictions and implemented the final model in a web-based research calculator. The findings may provide a basis for future external validation and prospective evaluation and may ultimately support oncology nurses in identifying high-risk patients, facilitating individualized antiemetic prophylaxis, monitoring modifiable predictors, initiating early preventive care, and delivering personalized symptom management after further validation.

## Methods

### Study design

This retrospective cohort study comprised three primary stages: the screening and selection of predictive variables, the development and internal validation of multiple prediction models, and the interpretation and visualization of the optimal model for clinical application. The study was reported in accordance with the Transparent Reporting of a Multivariable Prediction Model for Individual Prognosis or Diagnosis plus Artificial Intelligence (TRIPOD + AI) statement.^17^ Before starting the analyses, the study protocol was approved by the ethics committee of the participating hospital (approval number: BC20241234), and the study was conducted in accordance with the principles of the Declaration of Helsinki. We also registered this study in the Chinese Clinical Trial Registry (ChiCTR) (ChiCTR-2500110585). Due to the retrospective nature of this study, the requirement for written informed consent was waived.

### Source of data and participants

In this development and internal validation modelling study, participants were enrolled using convenience sampling. Between January 1, 2025 and December 31, 2025, a total of 1062 patients with liver cancer who received intra-arterial therapy at a cancer specialty hospital in mainland China were included. All data required for model development were extracted from questionnaires and the electronic medical record system, and only anonymous data were used in the analysis. The inclusion criteria were as follows: (1) patients aged 18 years or older with histological and pathological proof of primary liver cancer; (2) Child-Pugh class A or B and Eastern Cooperative Oncology Group (ECOG) performance score of < 2; (3) the decision for intra-arterial therapy, including drug-eluting bead transarterial chemoembolization or hepatic arterial infusion chemotherapy, was made by a formal multidisciplinary team meeting based on abdominal contrast-enhanced CT. We excluded patients if they had any of the following at study entry: (1) combined with other malignancies; (2) treated with transcatheter arterial embolization (TAE), defined as bland arterial embolization without intra-arterial chemotherapeutic drug administration; (3) received both TACE and HAIC during the same hospitalization or had a history of alternating TACE and HAIC across previous intra-arterial treatment sessions; (4) patients with ≥ 20% missing clinical data or incomplete 24-h postprocedural ACINV assessment records; (5) central nervous system disease or other primary diseases that can lead to nausea and vomiting. The index treatment was defined as the intra-arterial interventional procedure performed during the hospitalization from which the 24-h postprocedural ACINV outcome was assessed. Treatment type was classified according to the index treatment recorded in physician order records and interventional procedure records.

### Outcomes

In this study, ACINV was evaluated using version 5.0 of the Common Terminology Criteria for Adverse Events (CTCAE v5.0) published by the United States National Cancer Institute.^18^ The primary outcome was ACINV within 24 hours after intra-arterial interventional therapy. ACINV was defined as the occurrence of either CTCAE v5.0 grade ≥ 2 nausea or CTCAE v5.0 grade ≥ 2 vomiting; patients with both symptoms were also classified as having ACINV. According to the CTCAE v5.0, grade two nausea is defined as decreased oral intake without significant weight loss, dehydration or malnutrition. Grade two vomiting is defined as the need for outpatient intravenous hydration and medical intervention. Following intra-arterial interventional therapy, responsible ward oncology nurses assessed and documented nausea and vomiting during hourly rounds throughout the first 24 hours after treatment as part of routine postprocedural care. For patients with multiple symptom records, the highest documented CTCAE grade during this period was used for outcome classification. Outcome status was retrospectively determined from nursing assessment records and electronic medical records. Two independent outcome assessors, who were not involved in predictor extraction or model development, retrospectively classified ACINV outcomes using de-identified postprocedural nursing and medical records. The assessors were blinded to candidate predictor information and model outputs.

### Candidate predictors

Similar to systemic intravenous chemotherapy, intra-arterial interventional therapy (transarterial chemoembolization or hepatic arterial infusion chemotherapy) delivers chemotherapeutic agents into the patient's tumor-feeding arteries, causing ACINV. Therefore, we selected the candidate predictors associated with ACINV by systematic reviews and meta-analyses, focusing on predictors that are easily identifiable and readily available in clinical practice. Moreover, we referred to our previously published systematic review,^14^ which summarized the most common predictors of CINV. We removed, modified, and combined predictors based on expert judgement, identifying the following 18 potential candidate predictors for inclusion in this study: sex, age, body mass index, smoking history, alcohol consumption, history of morning sickness, history of motion sickness, existing comorbidity, stage of liver cancer, history of chemotherapy, history of ACINV, type of interventional therapy, interventional drug, history of intra-arterial interventional therapy, antiemetic regimen, anxiety, anticipatory nausea and vomiting, and sleep duration. These predictors encompass demographic characteristics, past medical history, tumor treatment history, intra-arterial interventional therapy factors, and psychological factors. Genetic markers were not included because they are not routinely assessed in clinical practice.

All candidate predictors were required to be available before intra-arterial interventional therapy. Demographic characteristics, medical history, tumor-related variables, and other baseline clinical variables were extracted from electronic medical records. Anxiety, anticipatory nausea and vomiting, and sleep duration were assessed on the morning of the procedure using preprocedural nursing assessment records. Anxiety was assessed using the Generalized Anxiety Disorder-7 scale (GAD-7),^19^ a standardized seven-item instrument. Each item is rated from 0 to 3, and the total score ranges from 0 to 21. A GAD-7 score of ≥ 5 was defined as anxiety. Anticipatory nausea and vomiting was assessed using a structured single-item nursing assessment: “Before the start of this interventional therapy, do you currently feel nausea or have vomiting in anticipation of the upcoming treatment?” The response categories were “yes” and “no”; patients reporting either nausea or vomiting were coded as “yes.” Sleep duration was obtained by directly asking patients about their total sleep time during the night before the procedure and was categorized as < 7 hours or ≥ 7 hours. These assessments were performed and documented by responsible ward oncology nurses who routinely conducted preprocedural nursing assessments. The type of intra-arterial interventional therapy, interventional drug, and antiemetic regimen were obtained from physician order records on the morning of the procedure before treatment initiation. In this study, the TACE group included only drug-eluting bead transarterial chemoembolization, and conventional TACE was not included. HAIC was defined as hepatic arterial infusion chemotherapy administered through an intra-arterial catheter without embolization. Notably, antiemetic regimen was classified according to the number and classes of prophylactic antiemetic drugs prescribed before treatment initiation. A single-agent regimen was defined as the use of one antiemetic drug, most commonly a 5-hydroxytryptamine-3 (5-HT3) receptor antagonist, including palonosetron, ondansetron, granisetron, or tropisetron. A regimen of two or more drugs was defined as the combined use of at least two antiemetic agents or drug classes, mainly a 5-HT3 receptor antagonist plus dexamethasone, with or without a neurokinin-1 (NK-1) receptor antagonist such as aprepitant or fosaprepitant.

### Sample size

The sample size was estimated using the pmsampsize package (parameters = 18, shrinkage = 0.9, C-statistic = 0.850, prevalence = 0.35) in R software.^20^ This package provides guidance on how to calculate the sample size required to develop clinical prediction models. The calculated minimum sample size required for this study was 404.

### Missing data assessment and data splitting

Missingness was assessed at both the predictor and patient levels before model development. Candidate predictors with more than 20% missing values and patients with missing values in more than 20% of candidate predictors were excluded from the analysis. The remaining derivation cohort was then randomly divided into a training set (60%) and an internal validation set (40%) using stratified sampling to preserve the distribution of ACINV outcomes. Data splitting was performed before any data-adaptive procedures, including imputation, feature transformation, predictor selection, class-imbalance handling, hyperparameter optimization, or model fitting.

Missing predictors were handled using a training-partition-specific MissForest procedure.^21^ Within each resampling iteration, MissForest was fitted using only the corresponding analysis partition, with ACINV status excluded from imputation. MissForest was used to complete the predictors in that analysis partition. Separate auxiliary random forest imputation models were then fitted for each predictor using the other completed predictors as inputs. When the fitted imputation procedure was applied to the corresponding assessment partition, observed values were retained unchanged. Missing values were predicted using the fixed auxiliary random forest models fitted to the completed analysis partition. No model was refitted using the assessment data. This procedure was repeated independently within every least absolute shrinkage and selection operator (LASSO), inner, and outer resampling split.

For final model development, MissForest was fitted to the complete training set. Auxiliary random forest models were then fitted to the MissForest-completed training predictors. These fixed models were applied, without refitting, to missing predictors in the held-out internal-validation set. Internal-validation outcomes were never used for imputation, preprocessing, predictor selection, model tuning, threshold selection, or algorithm selection.

### Data processing and feature selection

Categorical variables were transformed into dummy variables using one-hot encoding, and continuous variables were standardized using Z-score normalization. Within each resampling iteration, the category levels for one-hot encoding and the means and standard deviations for Z-score normalization were estimated exclusively from the corresponding training data and then applied unchanged to the held-out data, including the outer validation folds and the internal validation set, to prevent information leakage.

Predictor selection was performed using LASSO regression within the nested resampling framework.^22^ At every stage requiring predictor selection, a separate stratified five-fold LASSO procedure was conducted within the current analysis partition. Within each LASSO fold, MissForest-based imputation, auxiliary random forest imputation, one-hot encoding, and standardization were fitted using only the LASSO training subset. The fitted transformations were then applied unchanged to the corresponding LASSO assessment subset.

The penalty grid was expressed as fractions of the fold-specific maximum penalty. Binomial deviances were pooled across the five LASSO assessment folds, and the most regularized penalty within one standard error of the minimum deviance was selected. The preprocessing pipeline and LASSO model were then refitted using the complete current analysis partition. A source-level predictor was considered selected when at least one corresponding dummy–variable coefficient was nonzero. All prespecified levels of a selected categorical predictor were retained for downstream model fitting.

### Class imbalance handling

The synthetic minority over-sampling technique (SMOTE) was applied only after LASSO selection and only to the current model-fitting partition. It was never applied to a LASSO assessment fold, an inner assessment fold, an outer assessment fold, or the held-out internal-validation set. Consequently, all reported assessment metrics were calculated using observations with the original outcome prevalence.

A prespecified sensitivity analysis repeated the complete pipeline without SMOTE. Pipelines with and without SMOTE were evaluated using the same leakage-controlled resampling principles. The corresponding discrimination, classification, Brier score, and calibration results are presented in Supplementary Table S1.

### Model development and evaluation

A total of seven machine learning algorithms, including logistic regression (LR), decision tree (DT), random forest (RF), extreme gradient boosting (XGBoost), support vector machine (SVM), naive bayes (NB), and k-nearest neighbor (KNN), were employed to develop models. These seven algorithms were selected to represent a spectrum of modeling paradigms, from simple parametric baselines to flexible nonlinear learners. LR and NB provide relatively simple baselines, DT captures threshold-based nonlinear splits, RF and XGBoost model nonlinear interactions through tree ensembles, SVM offers a margin-based classifier with flexible decision boundaries, and KNN is a local nonparametric method.

Candidate models were developed and compared using nested stratified five-fold cross-validation within the training set. The outer cross-validation loop was used to generate out-of-fold (OOF) predictions for unbiased estimation of model performance and comparison of candidate algorithms. Within each outer training partition, an inner stratified five-fold cross-validation loop was conducted for hyperparameter tuning. Inner-loop OOF predictions were used to determine the optimal probability threshold by maximizing the Youden index, thereby preventing information leakage from the outer validation partitions.

Candidate algorithms were ranked according to pooled outer five-fold OOF area under the curve (AUC) in the training set. The algorithm with the highest pooled OOF AUC was selected. When OOF AUC values were identical, the model with the lower pooled OOF Brier score was selected as the tie-breaker. Calibration and decision-curve analyses were prespecified as complementary assessments and were not used to override the primary ranking rule.

The selected modeling pipeline was subsequently refitted using the complete training set (*n* = 638) with the optimized hyperparameters. The probability threshold derived during model development was retained for classification in the held-out internal-validation set. Candidate-model comparison and model selection were performed exclusively within the training set; the internal-validation set was not used for algorithm ranking or model optimization.

Candidate algorithms were ranked according to pooled outer five-fold OOF AUC in the training set. Lower pooled OOF Brier score was used only if two algorithms had identical AUC values. Calibration and decision-curve analyses were complementary assessments and did not override the prespecified ranking rule. After RF was selected, the complete pipeline was refitted using all 638 training observations. The RF mtry parameter was tuned over values of 2, 4, 8, and 12. The final model used mtry = 12, 500 trees, and a minimum terminal node size of 5. The final classification threshold of 0.3876 was obtained from five-fold OOF predictions generated within the training set. The locked pipeline was evaluated once in the held-out 424-patient internal-validation set in the current reanalysis. Decision-curve analysis was performed using pooled outer-fold OOF predictions for candidate-model comparison and using the held-out internal-validation data for final RF evaluation. The hyperparameter search space and final parameter settings are summarized in Supplementary Table S2.

Model performance was evaluated in terms of discrimination, calibration, classification performance, and clinical utility. AUC values and their 95% confidence intervals (CIs) were estimated using the DeLong method. Accuracy, sensitivity, specificity, positive predictive value, and negative predictive value were reported with exact Clopper–Pearson 95% CIs. Calibration was assessed using the Brier score and logistic calibration models. Calibration intercepts and slopes were reported with Wald 95% CIs. An intercept of 0 and a slope of one indicated ideal calibration. Decision-curve analysis used pooled outer-fold OOF predictions for candidate-model comparison and the held-out internal-validation data for final evaluation.

### Model interpretation and web-based application

The SHAP method was employed to provide both global and local interpretability for the model.^23^ SHAP values were calculated using the fastshap R package for the locked RF model. The prediction wrapper returned the RF-predicted probability of ACINV. The SHAP background dataset comprised the 638 original training-set observations after application of the locked preprocessing pipeline. SHAP values for one-hot encoded variables were summed across their corresponding source-level predictors. Accordingly, the reported SHAP values represent additive contributions on the predicted-probability scale relative to the mean predicted probability in the training set. Positive and negative SHAP values indicate increases and decreases, respectively, in the predicted probability of ACINV. Furthermore, we utilized the Shiny package^24^ to integrate the selected predictors and final model into a research prototype web-based calculator (model version v2.0, deployed on September 16, 2026) for preliminary ACINV-IAIT risk estimation. The web-based calculator uses the same final model, preprocessing rules, predictor coding, and classification threshold as reported in this study.

### Statistical description

Patients’ characteristics were reported as mean (standard deviation [SD]) or median (interquartile range [IQR]) for continuous variables and *n* (%) for categorical variables. Categorical variables were compared using the Chi-squared test or Fisher exact test, and continuous variables were analyzed using Wilcoxon or Kruskal–Wallis test. A two-sided *P* value < 0.05 was considered statistically significant. All statistical analysis and calculations were performed using statistical software R version 4.4.2.

## Results

### Baseline characteristics

The flowchart of the study design is presented in Fig. 1. Among all the 1377 assessed patients in this study, 315 patients were excluded following the exclusion criteria. Of these, 27 patients were excluded because they had missing values for more than 20% of the 18 candidate predictors, corresponding to four or more missing variables per patient. Missing data was concentrated in preprocedural nursing assessment variables, particularly anxiety, anticipatory nausea and vomiting, and sleep duration. No retained predictor had more than 20% missing values. Missingness was 1.951% in the training set and 1.979% in the internal validation set. The distribution of missing data is provided in Supplementary Table S3.

**Fig. 1:**
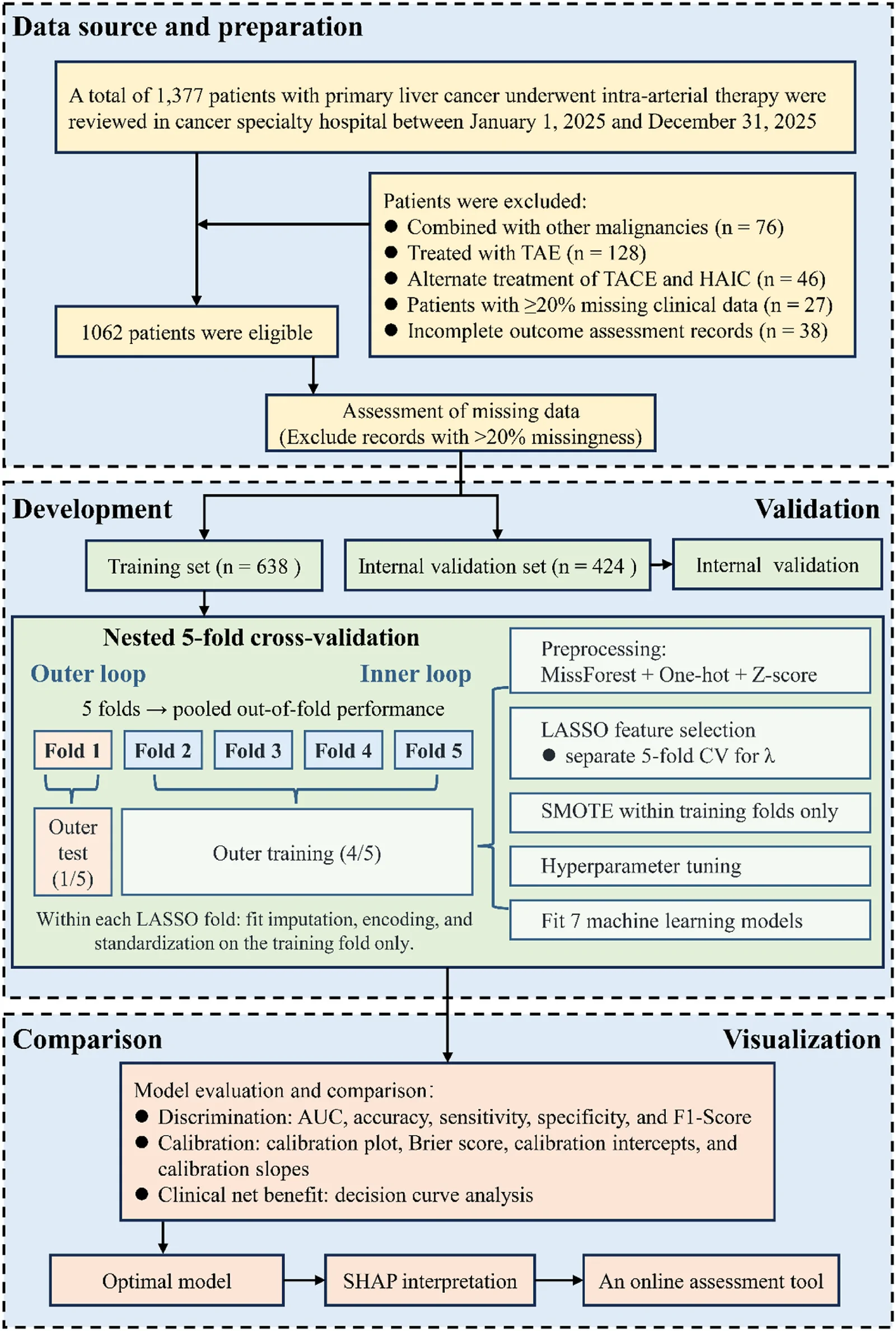
Flowchart of the study design. TAE, transcatheter arterial embolization; TACE, transarterial chemoembolization; HAIC, hepatic arterial infusion chemotherapy; LASSO, least absolute shrinkage and selection operator; SMOTE, synthetic minority over-sampling technique; AUC, area under the receiver operating characteristic curve; SHAP, Shapley additive explanations.

The remaining 1062 patients were randomly divided into a training set (*n* = 638) and an internal validation set (*n* = 424) in a 6:4 ratio. Differences in variable distributions between the training and internal validation sets, assessed using standardized mean differences (SMD), are presented in Supplementary Table S4. Most predictors showed small between-set differences; however, several variables had absolute SMDs exceeding 0.10, indicating modest distributional differences between the training and internal validation sets. In the entire cohort, the median age of the patients was 60 (IQR 53–67), 757 patients (71.3%) were men, and the median body mass index (BMI) was 23.51 (IQR 21.20–25.95). Baseline characteristics of patients with and without ACINV in the training and internal validation sets are shown in Table 1. According to the CTCAE v5.0, the incidence rates of ACINV were 35.4% and 36.3% in the training and internal validation sets, respectively. Univariate analyses showed that most predictors differed significantly between patients with and without ACINV, except for BMI, existing comorbidity, Barcelona Clinic Liver Cancer (BCLC) stage, history of chemotherapy, history of ACINV, and history of IAIT in the training set, and smoking history, existing comorbidity, and BCLC stage in the internal validation set.

**Table 1:** Baseline characteristics of the training set and internal validation set.

Characteristics	Training set (*n* = 638), *n* (%)		*P* value	Internal validation set (*n* = 424), *n* (%)		*P* value
	ACINV (*n* = 226)	Non-ACINV (*n* = 412)		ACINV (*n* = 154)	Non-ACINV (*n* = 270)	
Sex			< 0.001			< 0.001
Male	103 (45.6)	347 (84.2)		85 (55.2)	222 (82.2)	
Female	123 (54.4)	65 (15.8)		69 (44.8)	48 (17.8)	
Age (years)			< 0.001			< 0.001
< 50	50 (22.1)	80 (19.4)		44 (28.6)	40 (14.8)	
50-60	66 (29.2)	143 (34.7)		52 (33.8)	109 (40.4)	
61-70	87 (38.5)	108 (26.2)		48 (31.2)	65 (24.1)	
> 70	23 (10.2)	81 (19.7)		10 (6.5)	56 (20.7)	
BMI (kg/m²)			0.670			0.037
< 18.5	15 (6.6)	26 (6.3)		12 (7.8)	15 (5.6)	
18.5–23.9	113 (50.0)	210 (51.0)		80 (51.9)	127 (47.0)	
24.0–27.9	68 (30.1)	134 (32.5)		37 (24.0)	99 (36.7)	
> 27.9	30 (13.3)	42 (10.2)		25 (16.2)	29 (10.7)	
Smoking history			< 0.001			0.533
Yes	66 (29.2)	176 (42.7)		58 (37.7)	110 (40.7)	
No	160 (70.8)	236 (57.3)		96 (62.3)	160 (59.3)	
Alcohol consumption			< 0.001			0.005
Yes	48 (21.2)	252 (61.2)		43 (27.9)	112 (41.5)	
No	178 (78.8)	160 (38.8)		111 (72.1)	158 (58.5)	
History of morning sickness			< 0.001			< 0.001
Yes	65 (28.8)	22 (5.3)		52 (33.8)	48 (17.8)	
No	161 (71.2)	390 (94.7)		102 (66.2)	222 (82.2)	
History of motion sickness			0.005			0.023
Yes	63 (27.9)	75 (18.2)		35 (22.7)	38 (14.1)	
No	163 (72.1)	337 (81.8)		119 (77.3)	232 (85.9)	
Existing comorbidity			0.449			0.070
Yes	95 (42.0)	186 (45.1)		48 (31.2)	108 (40.0)	
No	131 (58.0)	226 (54.9)		106 (68.8)	162 (60.0)	
BCLC			0.499			0.451
A	27 (11.9)	45 (10.9)		18 (11.7)	22 (8.1)	
B	30 (13.3)	69 (16.7)		32 (20.8)	54 (20.0)	
C	169 (74.8)	298 (72.3)		104 (67.5)	194 (71.9)	
History of chemotherapy			0.333			< 0.001
Yes	79 (35.0)	160 (38.8)		33 (21.4)	105 (38.9)	
No	147 (65.0)	252 (61.2)		121 (78.6)	165 (61.1)	
History of ACINV			0.052			< 0.001
Yes	92 (40.7)	136 (33.0)		67 (43.5)	70 (25.9)	
No	134 (59.3)	276 (67.0)		87 (56.5)	200 (74.1)	
Type of interventional therapy			< 0.001			< 0.001
TACE	143 (63.3)	192 (46.6)		120 (77.9)	131 (48.5)	
HAIC	83 (36.7)	220 (53.4)		34 (22.1)	139 (51.5)	
Interventional drug			< 0.001			< 0.001
Platinum-based	164 (72.6)	194 (47.1)		115 (74.7)	118 (43.7)	
Non-platinum-based	62 (27.4)	218 (52.9)		39 (25.3)	152 (56.3)	
History of intra-arterial interventional therapy			0.610			0.007
Yes	83 (36.7)	143 (34.7)		36 (23.4)	97 (35.9)	
No	143 (63.3)	269 (65.3)		118 (76.6)	173 (64.1)	
Antiemetic regimen			< 0.001			< 0.001
Single-agent	102 (45.1)	115 (27.9)		79 (51.3)	79 (29.3)	
≥2 drugs	124 (54.9)	297 (72.1)		75 (48.7)	191 (70.7)	
Anxiety			< 0.001			< 0.001
Yes	170 (75.2)	116 (28.2)		110 (71.4)	79 (29.3)	
No	56 (24.8)	296 (71.8)		44 (28.6)	191 (70.7)	
Anticipatory nausea and vomiting			< 0.001			< 0.001
Yes	145 (64.2)	161 (39.1)		90 (58.4)	99 (36.7)	
No	81 (35.8)	251 (60.9)		64 (41.6)	171 (63.3)	
Sleep duration			< 0.001			< 0.001
< 7 hours	167 (73.9)	215 (52.2)		110 (71.4)	125 (46.3)	
≥ 7 hours	59 (26.1)	197 (47.8)		44 (28.6)	145 (53.7)	

BMI, body mass index; ACINV, acute chemotherapy-induced nausea and vomiting; BCLC, Barcelona Clinic Liver Cancer; TACE, transarterial chemoembolization; HAIC, hepatic arterial infusion chemotherapy.

### Predictor selection

All 18 prespecified candidate predictors were entered into the fold-specific LASSO procedure. Imputation, encoding, standardization, and LASSO penalty selection were repeated independently within the relevant training partitions. Outer-fold selection frequencies were reported as indicators of selection stability but were not used as eligibility criteria for the final model. After algorithm selection, the complete LASSO pipeline was refitted using all 638 training observations, with the penalty selected exclusively within the training set by the one-standard-error rule. The final penalty was *λ* = 0.01870. Predictors represented by at least one nonzero dummy–variable coefficient in this full-training-set refit were retained.

Ten predictors were selected in all five outer training folds. Smoking history was selected in two outer folds, whereas tumour stage was selected in one. In the final full-training-set refit, smoking history had a nonzero coefficient and was retained, whereas the coefficient for tumour stage was reduced to zero. The final pipeline therefore included 11 source-level predictors: sex, age, type of IAIT, interventional drug, history of ACINV, history of IAIT, sleep duration, antiemetic regimen, anticipatory nausea and vomiting, anxiety, and smoking history. Outer-fold selection frequencies are reported in Supplementary Table S5.

### Model development and performance comparison

Table 2 summarizes candidate-model performance based on pooled outer five-fold OOF predictions from the training set. RF achieved the highest OOF AUC, at 0.941 (95% CI, 0.923–0.958). XGBoost and SVM achieved AUCs of 0.933 (95% CI, 0.913–0.952) and 0.931 (95% CI, 0.912–0.951), respectively. RF also had the lowest Brier score, at 0.092. Its calibration intercept was −0.120 (95% CI, −0.371 to 0.132), and its calibration slope was 0.986 (95% CI, 0.825–1.147). Decision-curve analysis showed that RF provided positive net benefit across threshold probabilities from 0.05 to 0.80. Its net benefit was generally among the highest across the evaluated range. RF was therefore selected according to the prespecified pooled OOF AUC ranking rule. Pairwise DeLong comparisons based on the outer-fold OOF predictions are presented in Supplementary Table S6.

**Table 2:** Outer five-fold out-of-fold performance and calibration of candidate models in the training set.

Metric	LR	DT	RF	SVM	XGBoost	KNN	NB
**Discrimination and classification**							
AUC (95% CI)	0.865 (0.837–0.892)	0.893 (0.865–0.920)	0.941 (0.923–0.958)	0.931 (0.912–0.951)	0.933 (0.913–0.952)	0.913 (0.890–0.936)	0.853 (0.823–0.882)
ACC (95% CI)	0.770 (0.735–0.802)	0.842 (0.811–0.869)	0.875 (0.846–0.899)	0.864 (0.835–0.889)	0.867 (0.838–0.892)	0.837 (0.806–0.865)	0.740 (0.704–0.773)
SEN (95% CI)	0.867 (0.816–0.909)	0.872 (0.821–0.912)	0.907 (0.861–0.942)	0.876 (0.826–0.916)	0.867 (0.816–0.909)	0.881 (0.831–0.920)	0.832 (0.777–0.878)
SPE (95% CI)	0.716 (0.670–0.759)	0.825 (0.785–0.861)	0.857 (0.819–0.889)	0.857 (0.819–0.889)	0.867 (0.830–0.898)	0.813 (0.772–0.850)	0.689 (0.642–0.734)
PPV (95% CI)	0.626 (0.570–0.680)	0.732 (0.675–0.784)	0.777 (0.721–0.825)	0.770 (0.714–0.820)	0.781 (0.725–0.830)	0.721 (0.664–0.773)	0.595 (0.539–0.650)
NPV (95% CI)	0.908 (0.871–0.937)	0.921 (0.889–0.947)	0.944 (0.915–0.965)	0.927 (0.896–0.951)	0.922 (0.891–0.947)	0.925 (0.893–0.950)	0.882 (0.842–0.915)
F1 score	0.727	0.796	0.837	0.820	0.822	0.793	0.694
Brier score	0.151	0.123	0.092	0.100	0.095	0.123	0.193
**Calibration and threshold-related metrics**							
Calibration intercept (95% CI)	−0.523 (−0.730 to −0.316)	−0.363 (−0.583 to −0.143)	−0.120 (−0.371 to 0.132)	−0.101 (−0.351 to 0.150)	−0.141 (−0.392 to 0.111)	−0.456 (−0.664 to −0.248)	−0.553 (−0.756 to −0.349)
Calibration slope (95% CI)	0.824 (0.694–0.953)	0.664 (0.568–0.760)	0.986 (0.825–1.147)	0.803 (0.691–0.915)	0.798 (0.683–0.913)	0.367 (0.272–0.461)	0.261 (0.220–0.302)
Youden index	0.583	0.697	0.764	0.733	0.734	0.694	0.521
Median fold-specific Youden cutoff	0.359	0.338	0.365	0.330	0.310	0.519	0.193

LR, logistic regression; DT, decision tree; RF, random forest; SVM, support vector machine; XGBoost, extreme gradient boosting; KNN, k-nearest neighbor; NB, naive Bayes; AUC, area under the receiver operating characteristic curve; ACC, accuracy; SEN, sensitivity; SPE, specificity; PPV, positive predictive value; NPV, negative predictive value; CI, confidence interval.

Values were calculated from outer 5-fold out-of-fold predictions in the 638-patient training set. Preprocessing, LASSO feature selection, SMOTE, hyperparameter tuning and threshold selection were conducted within the corresponding resampling folds. AUC 95% CIs were calculated using the DeLong method. CIs for ACC, SEN, SPE, PPV and NPV were calculated using exact Clopper–Pearson intervals. Calibration intercepts and slopes were estimated using logistic calibration models. The locked internal-validation set was not used for candidate-model comparison.

The selected RF pipeline was refitted using the complete training set. The classification threshold of 0.3876 was locked before evaluation in the held-out internal-validation set. RF achieved an AUC of 0.962 (95% CI, 0.946–0.978), accuracy of 0.892 (95% CI, 0.858–0.919), and sensitivity of 0.942 (95% CI, 0.892–0.973). Its specificity was 0.863 (95% CI, 0.816–0.902), PPV was 0.797 (95% CI, 0.731–0.853), and NPV was 0.963 (95% CI, 0.931–0.983). The Brier score was 0.073. The calibration intercept was −0.090 (95% CI, −0.435 to 0.254), and the calibration slope was 1.056 (95% CI, 0.825–1.288) (Table 3).

**Table 3:** Internal-validation performance of the final RF model.

Metric	RF
AUC (95% CI)	0.962 (0.946–0.978)
ACC (95% CI)	0.892 (0.858–0.919)
SEN (95% CI)	0.942 (0.892–0.973)
SPE (95% CI)	0.863 (0.816–0.902)
PPV (95% CI)	0.797 (0.731–0.853)
NPV (95% CI)	0.963 (0.931–0.983)
F1 score	0.863
Brier score	0.073
Calibration intercept (95% CI)	−0.090 (−0.435 to 0.254)
Calibration slope (95% CI)	1.056 (0.825–1.288)
Youden index	0.805
Locked cutoff	0.3876

RF, random forest; AUC, area under the receiver operating characteristic curve; ACC, accuracy; SEN, sensitivity; SPE, specificity; PPV, positive predictive value; NPV, negative predictive value; CI, confidence interval.

At the locked threshold, the internal-validation confusion matrix comprised 233 true negatives, 37 false positives, nine false negatives, and 145 true positives (Supplementary Table S7). Exploratory risk strata were derived from tertiles of the pooled RF outer-fold OOF probabilities. Low risk was defined as a probability ≤ 0.041, intermediate risk as > 0.041 to ≤ 0.585, and high risk as > 0.585. Fig. 2 presents the outer-fold OOF ROC, calibration, and decision curves for all candidate models. It also presents the corresponding evaluation of the final RF model in the held-out internal-validation set.

**Fig. 2:**
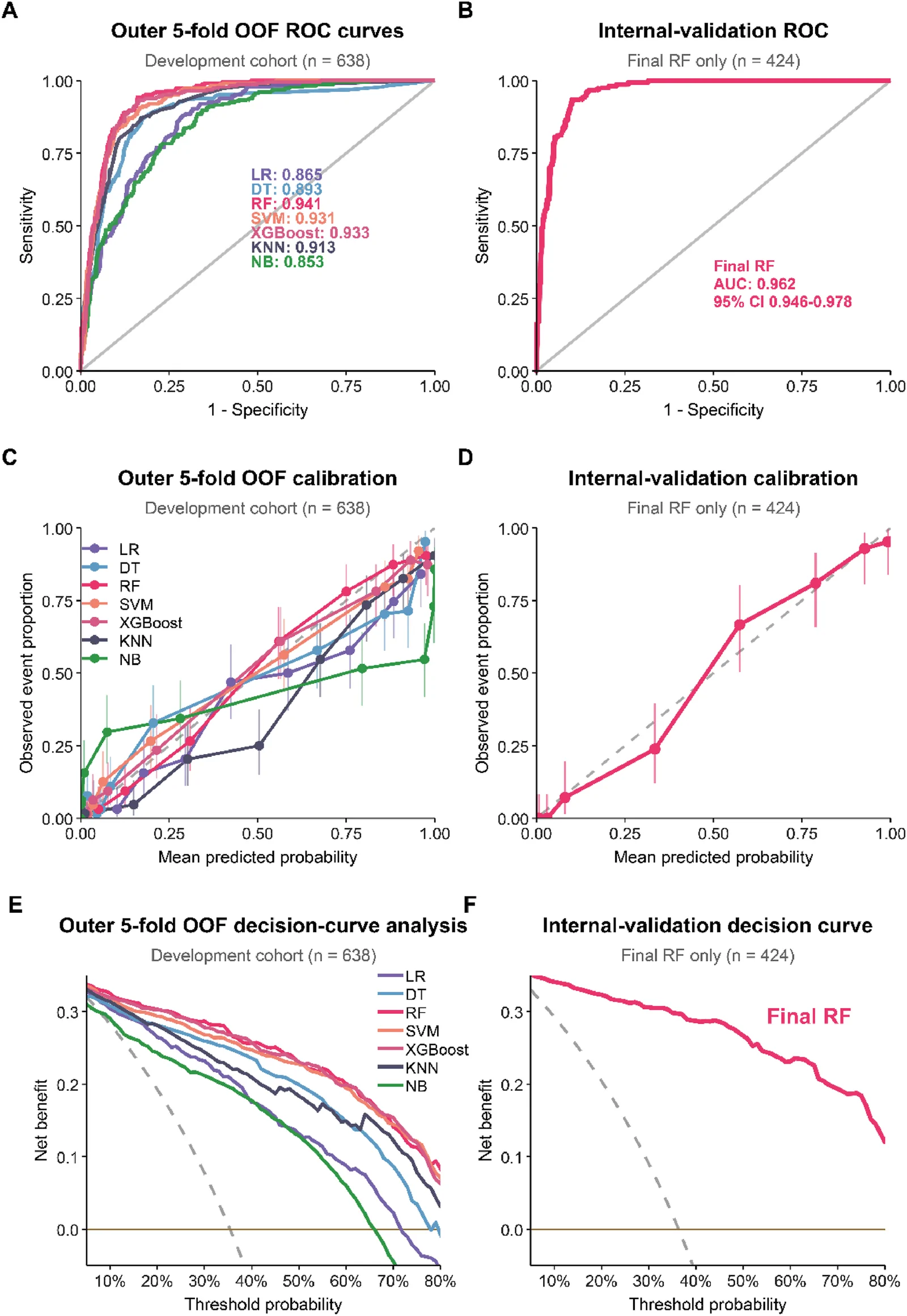
Receiver operating characteristic curves, calibration plots, and decision-curve analyses based on a nested five-fold cross-validation framework and locked internal validation. Panels (A), (C), and (E) show the ROC curves, calibration plots, and decision curves, respectively, for all seven candidate models using pooled outer five-fold out-of-fold predictions from the 638-patient training set. Panels (B), (D), and (F) show the corresponding results for the final RF model evaluated once in the locked 424-patient internal-validation cohort in the current reanalysis. ROC, receiver operating characteristic; OOF, out-of-fold; DCA, decision-curve analysis; LR, logistic regression; DT, decision tree; RF, random forest; SVM, support vector machine; XGBoost, extreme gradient boosting; KNN, k-nearest neighbor; NB, naive Bayes.

In the sensitivity analysis without SMOTE, RF remained the highest-ranked algorithm. Its pooled outer-fold OOF AUC was 0.942 (95% CI, 0.925–0.959), compared with 0.941 (95% CI, 0.923–0.958) for the primary pipeline with SMOTE. In internal validation, the RF pipeline without SMOTE achieved an AUC of 0.964 (95% CI, 0.947–0.980). However, its calibration slope was 1.604 and its Brier score was 0.076. The corresponding values for the primary pipeline were 1.056 and 0.073. The two pipelines therefore showed similar discrimination, whereas the primary pipeline showed more favorable internal-validation calibration (Supplementary Table S1).

### Model explanation and application

To elucidate the decision-making process of RF model, we employed the SHAP method to visualize the model's predictive outcomes. This explainable method provided global explanation of the model at the predictor level and local explanation at the individual level (Fig. 3) based on the SHAP values. The SHAP values represent each predictor's contribution to the RF-predicted ACINV probability. As shown in Fig. 3A, predictor importance was summarized using the mean absolute SHAP value on the predicted-probability scale. Fig. 3B shows that anxiety, sex, interventional drug, history of IAIT, type of IAIT, age, antiemetic regimen, sleep duration, history of ACINV, smoking history, and anticipatory nausea and vomiting contributed to the final RF predictions to varying degrees.

**Fig. 3:**
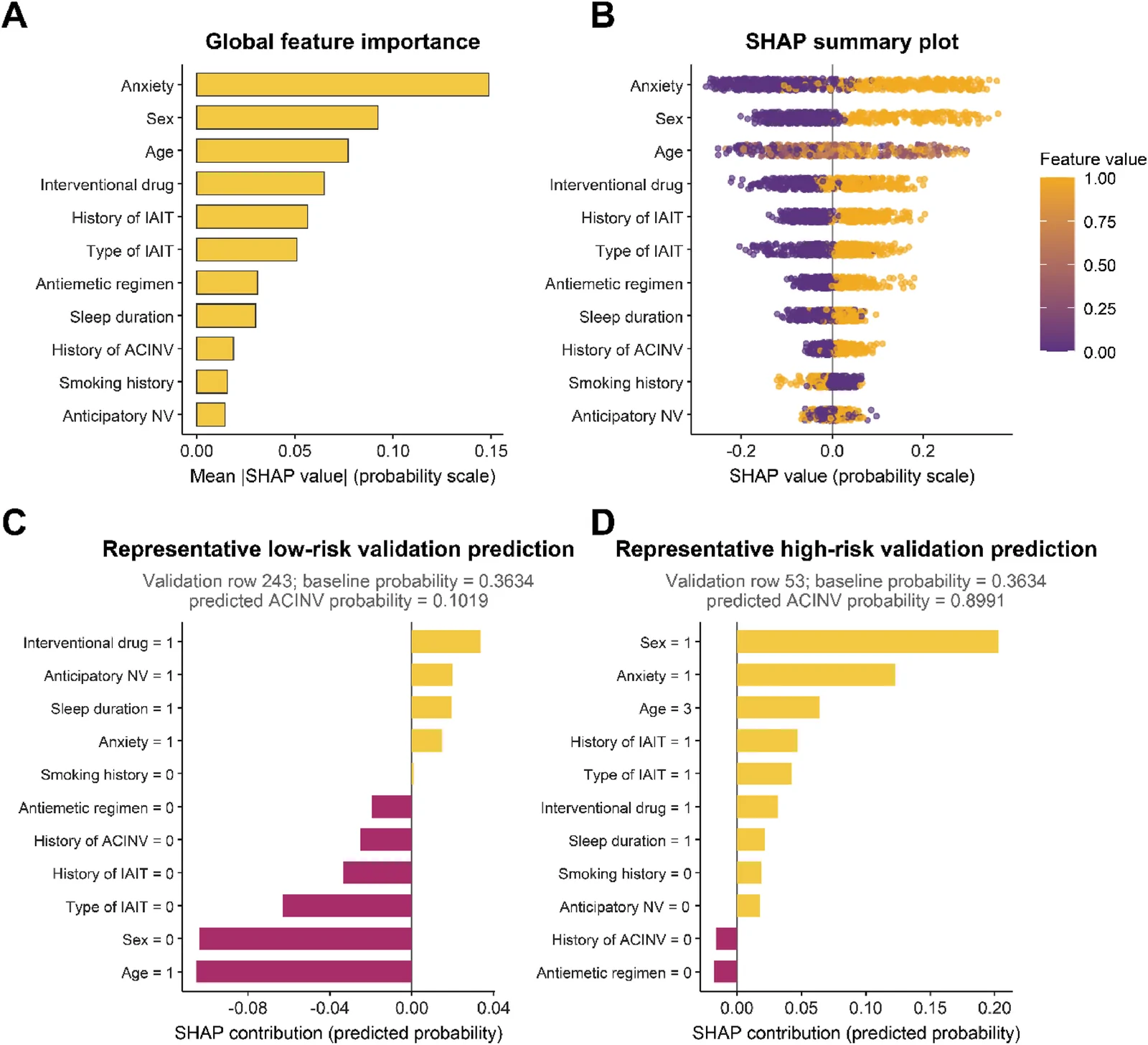
Global and local explanations of the RF model using probability-scale SHAP values (A) Ranking of predictor importance according to the mean absolute SHAP value in the development cohort. SHAP values were calculated using the fastshap R package, with the RF-predicted probability of ACINV as the model output (B) SHAP summary plot showing the distribution of source-level predictor contributions across the 638 patients in the development cohort. Each dot represents one patient. The horizontal position indicates the SHAP contribution on the predicted-probability scale. Positive SHAP values indicate an increase in the predicted ACINV probability, whereas negative SHAP values indicate a decrease. The color gradient represents the encoded predictor value, with purple indicating lower values and orange indicating higher values. Vertical jittering illustrates the density of observations (C, D) Local SHAP explanations for representative patients from the held-out internal-validation cohort classified as non-ACINV and ACINV, respectively. The predicted ACINV probabilities were 0.1019 and 0.8991, respectively. E [p(X)] represents the baseline mean predicted probability in the development cohort, and p(x) represents the patient-specific predicted probability. Gold bars indicate positive predictor contributions that increase the predicted ACINV probability, whereas purple bars indicate negative contributions that decrease it. Bar length represents the magnitude of each contribution on the predicted-probability scale. No logit or sigmoid transformation was applied to the reported SHAP values. RF, random forest; SHAP, Shapley additive explanations; IAIT, intra-arterial interventional therapy; ACINV, acute chemotherapy-induced nausea and vomiting; NV, nausea and vomiting. For categorical predictors, numerical codes were defined as follows: sex (male = 0, female = 1); age (< 50 years = 0, 50–60 years = 1, 61–70 years = 2, > 70 years = 3); type of interventional therapy (HAIC = 0, TACE = 1); interventional drug (non-platinum-based = 0, platinum-based = 1); history of ACINV (no = 0, yes = 1); history of intra-arterial interventional therapy (yes = 0, no previous IAIT = 1); antiemetic regimen (≥ 2 drugs = 0, single-agent = 1); anxiety (no = 0, yes = 1); anticipatory nausea and vomiting (no = 0, yes = 1); sleep duration (≥ 7 hours = 0, < 7 hours = 1); and smoking history (no = 0, yes = 1).

Additionally, two representative patients from the held-out internal-validation cohort were used to illustrate the local explanations (Fig. 3C and D). The RF-predicted ACINV probabilities were 0.1019 for the non-ACINV example and 0.8991 for the ACINV example. The baseline model output was the mean predicted probability in the development cohort, 0.3634. Gold bars represent positive SHAP contributions that increase the predicted ACINV probability, whereas purple bars represent negative contributions that decrease it. The magnitude of each bar represents the contribution on the predicted-probability scale. These SHAP values are model attributions and should not be interpreted as odds ratios or causal effects.

To facilitate interpretation and future evaluation of the ACINV-IAIT model by oncology nurses, we created a user-friendly risk assessment tool (model version v2.0, deployed on September 16, 2026) and deployed it on the Internet using the Shiny package, as shown in Fig. 4. The web-based calculator uses the same final RF model, selected predictors, preprocessing parameters, and variable coding scheme as reported in the manuscript. The web tool can be accessed online at the following link: https://yongjianwang.shinyapps.io/ACINV-IAIT/. These options are used to standardize user input and are not intended as default substitutions for missing values. All predictor fields must be completed according to the patient's actual clinical information before prediction. If any predictor value is unavailable or missing, the calculator should not be used to generate a risk estimate. Although complete input is required, all 11 predictors are routinely available before intra-arterial interventional therapy from electronic medical records, physician order records, and preprocedural nursing assessments. By inputting clinical 11 predictors directly into designated text fields on the webpage, oncology nurses can easily obtain both global and local prediction outcomes in individual patients with liver cancer.

**Fig. 4:**
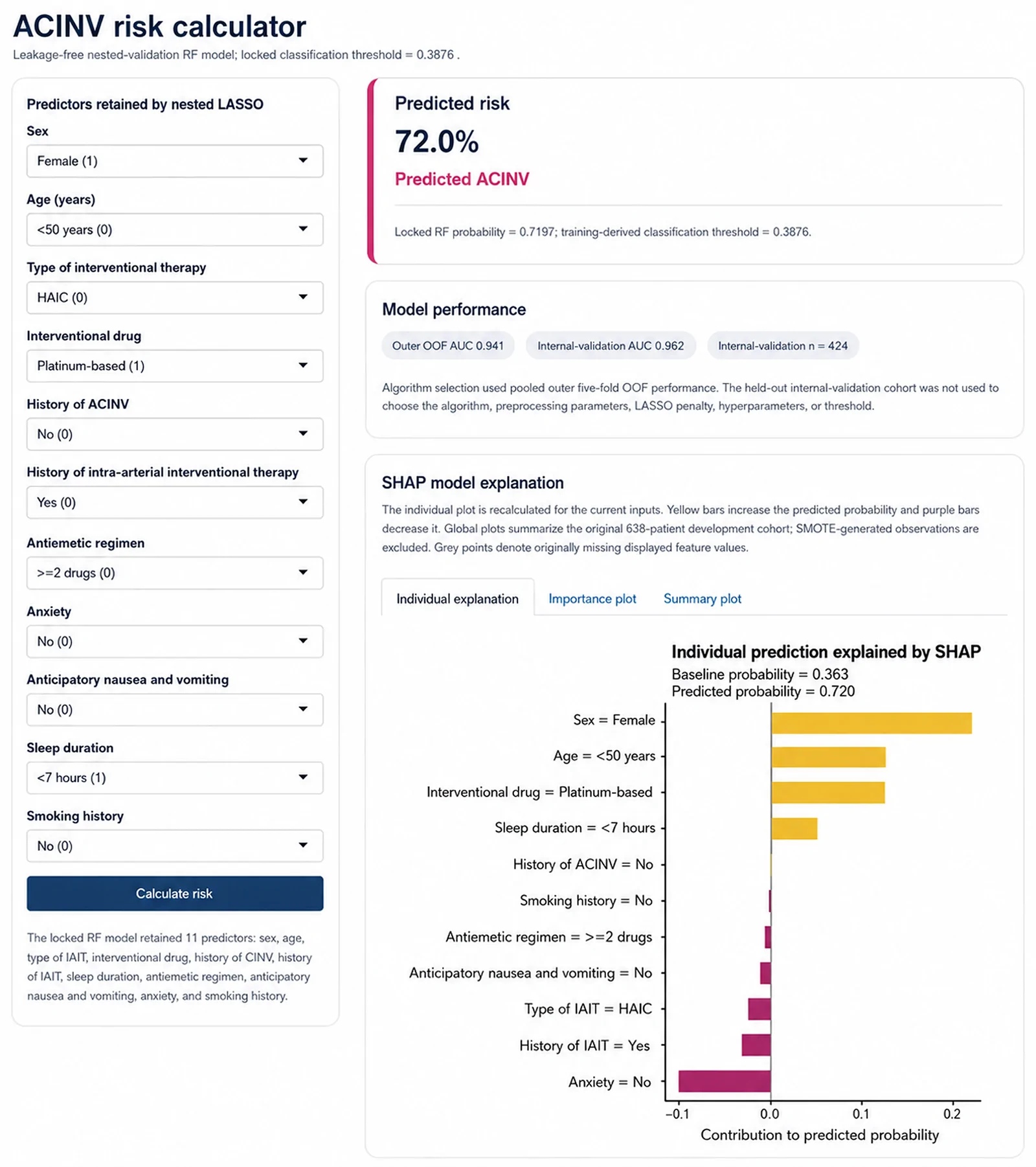
Web-based calculator for ACINV-IAIT risk assessment in patients with liver cancer. After all 11 predictors required by the final RF model have been entered according to the patient's actual clinical information, the calculator estimates the probability of ACINV-IAIT and provides probability-scale SHAP explanations. ACINV, acute chemotherapy-induced nausea and vomiting; HAIC, hepatic arterial infusion chemotherapy; IAIT, intra-arterial interventional therapy; LASSO, least absolute shrinkage and selection operator; RF, random forest; AUC, area under the receiver operating characteristic curve; OOF, out-of-fold; SHAP, Shapley additive explanations; SMOTE, synthetic minority over-sampling technique.

## Discussion

### Main findings

To our knowledge, this is the first study to develop and internally validate an interpretable machine-learning model for predicting ACINV-IAIT in patients with primary liver cancer. Seven candidate algorithms were compared using pooled outer-fold OOF predictions from a leakage-controlled nested resampling framework. RF achieved the highest OOF AUC, at 0.941 (95% CI, 0.923–0.958). The locked RF model achieved an AUC of 0.962 (95% CI, 0.946–0.978) in the held-out internal-validation set. Its Brier score was 0.073, calibration intercept was −0.090, and calibration slope was 1.056. Calibration plots suggested acceptable calibration, and decision curve analysis indicated potential net benefit across a range of threshold probabilities. Probability-scale SHAP analysis was used to visualize the contribution of individual predictors to the RF-predicted ACINV probability and to improve model interpretability. We also developed a prototype online risk assessment tool to support future clinical evaluation. Overall, this model may help identify patients at increased risk of ACINV-IAIT before interventional therapy, thereby supporting oncology nursing teams in planning individualized supportive care strategies.

The identification of key predictors associated with ACINV-IAIT significantly contributes to the risk assessment and individualized supportive care. This study identified 11 clinically available predictors, including treatment-related factors (type of interventional therapy, interventional drug, history of interventional therapy, and antiemetic regimen), patient-related factors (sex, age, history of ACINV, sleep duration, and smoking history), and psychological and emotional factors (anxiety and anticipatory nausea and vomiting). Among these variables, anxiety had the highest mean absolute SHAP value on the predicted-probability scale in the global explanation.

Anxiety in patients with liver cancer frequently manifests as panic, irritability, and pervasive nervousness, often linked to concerns regarding disease prognosis and the effects of interventional chemotherapy. The research^25^ suggested that anxiety activates the hypothalamic–pituitary–adrenal axis and sympathetic nervous system, leading to elevated cortisol and norepinephrine levels. These stress hormones may sensitize the chemoreceptor trigger zone and vagal afferent pathways, amplifying the nausea and vomiting response to chemotherapeutic agents. This result illustrates the importance of psychological assessment before interventional treatment in patients with liver cancer.

Consistent with many previous studies,^26^^,^^27^ female sex, interventional therapy with platinum-based drugs, younger age, use of a single antiemetic drug, short sleep duration (< 7 hours), history of ACINV, smoking history, and anticipatory nausea and vomiting before interventional therapy were predictors for ACINV after interventional therapy. These findings further support the applicability of established ACINV predictors in patients undergoing intra-arterial interventional treatment for liver cancer.

Notably, our study revealed that patients with liver cancer who received interventional treatment for the first time exhibited a higher risk of ACINV. Patients undergoing interventional therapy for the first time may experience heightened negative emotions or stress about the unknown side effects of interventional therapy, which can exacerbate the perception and severity of ACINV.^28^ Furthermore, the initial arterial infusion or chemoembolization may heighten sensitivity in both the gastrointestinal and central nervous systems, thereby increasing susceptibility to ACINV.^29^^,^^30^ However, patients who have undergone multiple treatments may have developed some degree of tolerance and adaptive resistance over time.

Another result was that patients who underwent TACE were associated with a higher risk of ACINV compared to those who received HAIC. These two intra-arterial treatments differ substantially in drug delivery, embolization, ischemic injury, inflammatory response, and potential emetogenic mechanisms. Drug-eluting bead TACE delivers chemotherapeutic agents locally through drug-loaded embolic beads and induces arterial embolization, which may lead to tumor ischemia, local tissue injury, inflammatory mediator release, and postembolization symptoms.^5^ These processes may stimulate visceral afferent pathways and contribute to acute nausea and vomiting.^31^ By contrast, HAIC delivers chemotherapeutic agents through continuous intra-arterial infusion without embolization, resulting in a different exposure pattern and less embolization-related ischemic injury.^32^ Oncology nursing teams should consider the interventional procedure when planning symptom monitoring and preventive care, with particular attention to patients undergoing TACE.

To date, numerous studies have focused on predicting ACINV in patients undergoing systemic chemotherapy, but there is a lack of a predictive model for ACINV-IAIT in patients with liver cancer.^11^, ^12^, ^13^ Although two existing CINV risk prediction tools developed for patients receiving intravenous chemotherapy were previously applied to assess the risk of delayed chemotherapy-induced nausea and vomiting after TACE, their applicability to ACINV-IAIT remains uncertain.^33^ Moreover, many existing studies have constructed models using a single method or relied on relatively small sample sizes, which may affect predictive performance. In this study, we applied seven machine-learning algorithms to predict ACINV-IAIT in patients with liver cancer. In the present comparison, RF achieved the highest prespecified pooled OOF AUC among seven algorithms. It also showed favorable Brier scores and calibration in the training-set OOF and internal-validation analyses. The similar discrimination obtained without SMOTE supported the stability of the algorithm selection. These findings support RF as the final algorithm for subsequent external validation. By applying this risk prediction model, oncology nurses may benefit from a practical and efficient tool for quickly identifying high-risk patients and predictors associated with ACINV. After further external validation, this tool may support earlier identification of patients at higher predicted risk and help oncology nurses plan individualized supportive care, symptom monitoring, and communication with physicians regarding prophylactic antiemetic strategies.

To interpret the RF model comprehensively and visually present the predictive results to oncology nurses, we applied the SHAP method to provide global and local explanations^34^^,^^35^ and designed an online risk assessment tool for ACINV-IAIT. Global SHAP analysis showed the relative contribution of each predictor, whereas local SHAP analysis illustrated how patient-specific characteristics contributed to individualized risk estimates.^36^ SHAP values quantified each predictor's contribution to the RF-predicted probability of ACINV, providing individualized explanations of the factors contributing to each patient's predicted risk and thereby improving the interpretability of the model. Overall, by using the SHAP-interpreted online ACINV-IAIT risk assessment tool, we gained insight into the internal decision-making mechanisms of the RF model and improved model interpretability and may enhance future clinical applicability after external validation.

### Implications for nursing practice and research

In clinical practice, oncology nurses could input readily available predictors into the ACINV-IAIT online tool to generate individualized ACINV risk for patients before interventional therapy. By visualizing the SHAP values for each predictor, oncology nurses were able to observe the direction and magnitude of their impact on the prediction of ACINV. For example, predictors such as female sex and a history of chemotherapy-induced nausea and vomiting made positive contributions to the model-predicted probability of ACINV, whereas antiemetic regimen and sleep duration of ≥ 7 hours made negative contributions. These explanations may facilitate a more individualized understanding of patient-specific risk profiles, helping oncology nurses interpret the basis of risk stratification and translate model outputs into clinically meaningful nursing actions during future clinical evaluation.

For patients classified as high risk, oncology nursing teams could provide more targeted supportive care, including psychological assessment, screening for anticipatory nausea and vomiting, individualized patient education, sleep support, relaxation training, and strategies to reduce anticipatory nausea. Risk stratification may also support communication with physicians and pharmacists before prophylactic antiemetic strategies are finalized. After treatment, high-risk patients could receive closer observation during the first 24 hours, earlier symptom assessment, timely rescue antiemetic management, hydration support, and reinforced education on reporting nausea or vomiting. Future nursing research should evaluate whether integrating this model into oncology nursing workflows improves symptom assessment, intervention timeliness, patient education, and patient-reported outcomes.

### Limitations

This study has several limitations. First, the model was developed and evaluated at a single centre in China. Its transportability to institutions with different patient characteristics, IAIT practices, antiemetic protocols, and liver cancer aetiologies remains uncertain. Although the internal-validation cohort was fully isolated from model development in the current reanalysis, it had been used for model evaluation in previous manuscript versions. Therefore, these estimates require cautious interpretation and confirmation through independent external validation before clinical implementation. Second, antiemetic regimen may partly reflect clinicians’ pre-existing assessment of patient risk. Consequently, its predictive contribution may be affected by confounding by indication and should not be interpreted as a causal treatment effect. Third, the study assessed ACINV within 24 hours after IAIT and did not evaluate delayed nausea and vomiting, biomarkers, genetic susceptibility, or patient-reported outcomes. Finally, the web-based calculator has not undergone prospective clinical impact evaluation. Its ability to improve care processes, symptom burden, or patient outcomes remains to be determined.

## Conclusions

In conclusion, we developed an interpretable RF-based risk prediction model for ACINV-IAIT in patients with primary liver cancer and evaluated its performance in an internal validation set. The model showed promising discrimination and calibration and may facilitate preliminary risk stratification before intra-arterial interventional therapy. By identifying patients at higher risk of ACINV, the model may help oncology nurses focus on key predictors, provide early warning, strengthen psychological assessment and sleep management, initiate preventive supportive care, and improve postprocedural symptom monitoring. To improve accessibility, the model was integrated into a web-based application for preliminary risk assessment. However, multicenter external validation and prospective impact studies are required before routine clinical implementation. After further validation, this tool may contribute to individualized oncology nursing care, reduced symptom burden, and improved quality of life in patients with liver cancer.

## CRediT authorship contribution statement

**Yan Lu:** Writing – original draft, Writing – review & editing, Conceptualization, Data curation, Formal analysis, Methodology, Visualization, Funding acquisition. **Ruishuang Zheng:** Writing – review & editing, Conceptualization, Data curation, Formal analysis, Methodology, Visualization, Funding acquisition. **Haipeng Yu:** Writing – review & editing, Supervision, Methodology, Formal analysis, Conceptualization. **Xueling Yang:** Formal analysis, Data curation, Methodology, Conceptualization. **Yanan Liu:** Formal analysis, Data curation, Validation. **Yongjian Wang:** Data curation, Methodology, Supervision, Conceptualization, Investigation, Funding acquisition. **Dongjun Liu:** Methodology, Supervision, Conceptualization, Investigation, Funding acquisition. All authors have read and approved the final manuscript.

## Ethics statement

The study was approved by the Medical Ethics Committee of Tianjin Medical University Cancer Hospital and Institute (Approval No. BC20241234) and was conducted in accordance with the 1964 Helsinki Declaration and its later amendments or comparable ethical standards. The requirement for written informed consent was waived by the ethics committee because of the retrospective nature of the study.

## Data availability

The data that support the findings of this study are available on request from the corresponding authors, YW and DL. The data are not publicly available because they contain information that could compromise the privacy of the research participants.

## Declaration of generative AI and AI-assisted technologies in the writing process

No generative AI or AI-assisted technologies were used in the preparation of this work.

## Funding

This research project was supported by the Nursing Special Fund for Tianjin Medical University Cancer Hospital and Institute (Grant No. H2303) and Tianjin Key Medical Discipline Construction Project (Grant No. TJYXZDXK-3-003 A). The funders had no role in the study design; data collection, analysis, or interpretation; preparation of the manuscript; or the decision to submit the article for publication.

## Declaration of competing interest

The authors declare no conflict of interest. The author, Dr. Ruishuang Zheng, is an editorial board member of Asia–Pacific Journal of Oncology Nursing. The article was subject to the journal's standard procedures, with peer review handled independently of Dr. Zheng and the research groups.
