# Supplementary material for: Development and internal validation of a machine learning model for predicting acute chemotherapy-induced nausea and vomiting after intra-arterial interventional therapy in patients with liver cancer
Source: Asia Pac J Oncol Nurs. 2026 Oct 3;13:101040. doi: 10.1016/j.apjon.2026.101040 (PMC13769129; doi:10.1016/j.apjon.2026.101040)
Supplement: Supplementary file 1 [file mmc1.docx]

**Table S1. Sensitivity analysis comparing models developed with and without SMOTE using outer five-fold out-of-fold predictions in the training set.**

| **Metric** | **LR** | **DT** | **RF** | **SVM** | **XGBoost** | **KNN** | **NB** |
| --- | --- | --- | --- | --- | --- | --- | --- |
| **Without SMOTE** | | | | | | | |
| AUC (95% CI) | 0.867 (0.840–0.894) | 0.890 (0.862–0.917) | 0.942 (0.925–0.959) | 0.925 (0.904–0.946) | 0.936 (0.918–0.955) | 0.912 (0.889–0.935) | 0.858 (0.829–0.886) |
| ACC (95% CI) | 0.754 (0.719–0.787) | 0.850 (0.819–0.876) | 0.879 (0.851–0.904) | 0.864 (0.835–0.889) | 0.864 (0.835–0.889) | 0.845 (0.814–0.872) | 0.738 (0.702–0.772) |
| SEN (95% CI) | 0.894 (0.846–0.931) | 0.885 (0.836–0.923) | 0.920 (0.877–0.952) | 0.863 (0.811–0.905) | 0.898 (0.851–0.934) | 0.907 (0.861–0.942) | 0.850 (0.796–0.894) |
| SPE (95% CI) | 0.677 (0.630–0.722) | 0.830 (0.790–0.865) | 0.857 (0.819–0.889) | 0.864 (0.827–0.896) | 0.845 (0.806–0.878) | 0.811 (0.769–0.847) | 0.677 (0.630–0.722) |
| PPV (95% CI) | 0.603 (0.548–0.656) | 0.741 (0.684–0.792) | 0.779 (0.724–0.827) | 0.777 (0.720–0.827) | 0.760 (0.704–0.810) | 0.724 (0.668–0.776) | 0.591 (0.535–0.645) |
| NPV (95% CI) | 0.921 (0.884–0.949) | 0.929 (0.898–0.953) | 0.951 (0.924–0.971) | 0.920 (0.888–0.945) | 0.938 (0.908–0.960) | 0.941 (0.911–0.963) | 0.891 (0.852–0.924) |
| F1 score | 0.720 | 0.806 | 0.844 | 0.818 | 0.824 | 0.806 | 0.697 |
| Brier score | 0.146 | 0.122 | 0.090 | 0.099 | 0.093 | 0.112 | 0.187 |
| Calibration intercept (95% CI) | −0.022 (−0.238–0.194) | −0.137 (−0.352–0.078) | −0.031 (−0.287–0.225) | −0.062 (−0.311–0.188) | −0.029 (−0.285–0.227) | −0.024 (−0.229–0.180) | −0.449 (−0.653 to −0.244) |
| Calibration slope (95% CI) | 0.905 (0.763–1.047) | 0.634 (0.540–0.728) | 1.068 (0.893–1.243) | 0.944 (0.814–1.074) | 0.856 (0.732–0.981) | 0.360 (0.269–0.451) | 0.280 (0.237–0.324) |
| Youden index | 0.571 | 0.715 | 0.777 | 0.727 | 0.743 | 0.718 | 0.527 |
| Median fold-specific Youden cutoff | 0.222 | 0.321 | 0.363 | 0.414 | 0.272 | 0.317 | 0.110 |
| **With SMOTE** | | | | | | | |
| AUC (95% CI) | 0.865 (0.837–0.892) | 0.893 (0.865–0.920) | 0.941 (0.923–0.958) | 0.931 (0.912–0.951) | 0.933 (0.913–0.952) | 0.913 (0.890–0.936) | 0.853 (0.823–0.882) |
| ACC (95% CI) | 0.770 (0.735–0.802) | 0.842 (0.811–0.869) | 0.875 (0.846–0.899) | 0.864 (0.835–0.889) | 0.867 (0.838–0.892) | 0.837 (0.806–0.865) | 0.740 (0.704–0.773) |
| SEN (95% CI) | 0.867 (0.816–0.909) | 0.872 (0.821–0.912) | 0.907 (0.861–0.942) | 0.876 (0.826–0.916) | 0.867 (0.816–0.909) | 0.881 (0.831–0.920) | 0.832 (0.777–0.878) |
| SPE (95% CI) | 0.716 (0.670–0.759) | 0.825 (0.785–0.861) | 0.857 (0.819–0.889) | 0.857 (0.819–0.889) | 0.867 (0.830–0.898) | 0.813 (0.772–0.850) | 0.689 (0.642–0.734) |
| PPV (95% CI) | 0.626 (0.570–0.680) | 0.732 (0.675–0.784) | 0.777 (0.721–0.825) | 0.770 (0.714–0.820) | 0.781 (0.725–0.830) | 0.721 (0.664–0.773) | 0.595 (0.539–0.650) |
| NPV (95% CI) | 0.908 (0.871–0.937) | 0.921 (0.889–0.947) | 0.944 (0.915–0.965) | 0.927 (0.896–0.951) | 0.922 (0.891–0.947) | 0.925 (0.893–0.950) | 0.882 (0.842–0.915) |
| F1 score | 0.727 | 0.796 | 0.837 | 0.820 | 0.822 | 0.793 | 0.694 |
| Brier score | 0.151 | 0.123 | 0.092 | 0.100 | 0.095 | 0.123 | 0.193 |
| Calibration intercept (95% CI) | −0.523 (−0.730 to −0.316) | −0.363 (−0.583 to −0.143) | −0.120 (−0.371–0.132) | −0.101 (−0.351–0.150) | −0.141 (−0.392–0.111) | −0.456 (−0.664 to −0.248) | −0.553 (−0.756 to −0.349) |
| Calibration slope (95% CI) | 0.824 (0.694–0.953) | 0.664 (0.568–0.760) | 0.986 (0.825–1.147) | 0.803 (0.691–0.915) | 0.798 (0.683–0.913) | 0.367 (0.272–0.461) | 0.261 (0.220–0.302) |
| Youden index | 0.583 | 0.697 | 0.764 | 0.733 | 0.734 | 0.694 | 0.521 |
| Median fold-specific Youden cutoff | 0.359 | 0.338 | 0.365 | 0.330 | 0.310 | 0.519 | 0.193 |

Abbreviations: LR, logistic regression; DT, decision tree; RF, random forest; SVM, support vector machine; XGBoost, extreme gradient boosting; KNN, k-nearest neighbor; NB, naive Bayes; AUC, area under the receiver operating characteristic curve; ACC, accuracy; SEN, sensitivity; SPE, specificity; PPV, positive predictive value; NPV, negative predictive value; CI, confidence interval.

### **Table S2. Hyperparameter search grids and selected hyperparameters from nested five-fold cross-validation in the primary analysis with SMOTE.**

| **Model** | **Algorithm and fixed settings** | **Hyperparameter search grid** | **Parameters selected across the five outer folds** | **Final locked model** |
| --- | --- | --- | --- | --- |
| LR | Ridge-penalized logistic regression; family = binomial; alpha = 0; standardize = FALSE | λ = 0.0001, 0.001, or 0.01 | λ = 0.01 in 5/5 folds | — |
| DT | Classification decision tree; minsplit = 20; xval = 0 | cp = 0.001, 0.01, or 0.05 | cp = 0.001 in 4/5 folds; cp = 0.01 in 1/5 fold | — |
| RF | Probability random forest; 500 trees; minimum terminal node size = 5 | mtry = 2, 4, 8, or 12 | mtry = 12 in 3/5 folds; mtry = 8 in 2/5 folds | mtry = 12; 500 trees; minimum terminal node size = 5 |
| SVM | Radial-basis-function kernel; probability = TRUE; scale = FALSE | cost = 0.5, 1, or 2; gamma = 0.01 or 0.05 | cost = 2 and gamma = 0.05 in 4/5 folds; cost = 1 and gamma = 0.05 in 1/5 fold | — |
| XGBoost | Binary logistic objective; min_child_weight = 1; subsample = 0.9; colsample_bytree = 0.9; nthread = 1 | max_depth = 2, 3, or 5; eta = 0.05 or 0.10; nrounds = 120 | max_depth = 5 and eta = 0.05 in 2/5 folds; max_depth = 5 and eta = 0.10 in 3/5 folds | — |
| KNN | Euclidean-distance KNN classifier | k = 5, 11, or 21 | k = 5 in 2/5 folds; k = 11 in 2/5 folds; k = 21 in 1/5 fold | — |
| NB | Naive Bayes classifier | Laplace = 0 or 1 | Laplace = 0 in 5/5 folds | — |

Abbreviations: LR, logistic regression; DT, decision tree; RF, random forest; SVM, support vector machine; XGBoost, extreme gradient boosting; KNN, k-nearest neighbor; NB, naive Bayes

**Table S3. Variable-level missingness and handling of missing data**

| **Predictor** | **Missing in excluded patients (*n* = 27), *n* (%)** | **Missing in included cohort (*n* = 1,062), *n* (%)** | **Training set (*n* = 638), *n* (%)** | **Internal validation set (*n* = 424), *n* (%)** | **Handling** |
| --- | --- | --- | --- | --- | --- |
| Sex | 0 (0.000) | 0 (0.000) | 0 (0.000) | 0 (0.000) | Complete |
| Age | 0 (0.000) | 0 (0.000) | 0 (0.000) | 0 (0.000) | Complete |
| Body mass index | 8 (29.630) | 20 (1.883) | 12 (1.881) | 8 (1.887) | MissForest imputation |
| Smoking history | 8 (29.630) | 28 (2.637) | 17 (2.665) | 11 (2.594) | MissForest imputation |
| Alcohol consumption | 7 (25.926) | 25 (2.354) | 15 (2.351) | 10 (2.358) | MissForest imputation |
| History of morning sickness | 6 (22.222) | 18 (1.695) | 11 (1.724) | 7 (1.651) | MissForest imputation |
| History of motion sickness | 6 (22.222) | 16 (1.507) | 9 (1.411) | 7 (1.651) | MissForest imputation |
| Existing comorbidity | 7 (25.926) | 22 (2.072) | 13 (2.038) | 9 (2.123) | MissForest imputation |
| BCLC stage | 4 (14.815) | 14 (1.318) | 8 (1.254) | 6 (1.415) | MissForest imputation |
| History of chemotherapy | 5 (18.519) | 21 (1.977) | 12 (1.881) | 9 (2.123) | MissForest imputation |
| History of ACINV | 6 (22.222) | 26 (2.448) | 16 (2.508) | 10 (2.358) | MissForest imputation |
| Type of interventional therapy | 0 (0.000) | 0 (0.000) | 0 (0.000) | 0 (0.000) | Complete |
| Interventional drug | 0 (0.000) | 0 (0.000) | 0 (0.000) | 0 (0.000) | Complete |
| History of intra-arterial interventional therapy | 3 (11.111) | 17 (1.601) | 10 (1.567) | 7 (1.651) | MissForest imputation |
| Antiemetic regimen | 0 (0.000) | 0 (0.000) | 0 (0.000) | 0 (0.000) | Complete |
| Anxiety | 22 (81.481) | 57 (5.367) | 34 (5.329) | 23 (5.425) | MissForest imputation |
| Anticipatory nausea and vomiting | 20 (74.074) | 51 (4.802) | 31 (4.859) | 20 (4.717) | MissForest imputation |
| Sleep duration | 22 (81.481) | 60 (5.650) | 36 (5.643) | 24 (5.660) | MissForest imputation |
| Total missing values | — | 375/19,116 (1.962) | 224/11,484 (1.951) | 151/7,632 (1.979) | — |

Abbreviations: BCLC, Barcelona Clinic Liver Cancer; ACINV: acute chemotherapy-induced nausea and vomiting. Counts in excluded patients are not mutually exclusive. All 27 excluded patients had missing values for more than 20% of the 18 candidate predictors. Missing values among included patients were imputed using MissForest.

**Table S4. Comparison of predictor distributions between the training and internal validation sets using standardized mean differences.**

| **Predictor** | **Training set (*n* = 638), *n* (%)** | **Internal validation set (*n* = 424), *n* (%)** | **Absolute SMD** |
| --- | --- | --- | --- |
| Sex, male | 450 (70.533) | 307 (72.406) | 0.041 |
| Age, years | <50: 130 (20.376);  50–60: 209 (32.759);  61–70: 195 (30.564);  >70: 104 (16.301) | <50: 84 (19.811);  50–60: 161 (37.972);  61–70: 113 (26.651);  >70: 66 (15.566) | 0.109 |
| Body mass index, kg/m² | <18.5: 41 (6.426);  18.5–23.9: 323 (50.627);  24.0–27.9: 202 (31.661);  >27.9: 72 (11.285) | <18.5: 27 (6.368);  18.5–23.9: 207 (48.821);  24.0–27.9: 136 (32.075);  >27.9: 54 (12.736) | 0.045 |
| Smoking history, yes | 242 (37.931) | 168 (39.623) | 0.035 |
| Alcohol consumption, yes | 300 (47.022) | 155 (36.557) | 0.213 |
| History of morning sickness, yes | 87 (13.636) | 100 (23.585) | 0.258 |
| BCLC stage | A: 72 (11.285);  B: 99 (15.517);  C: 467 (73.197) | A: 40 (9.434);  B: 86 (20.283);  C: 298 (70.283) | 0.125 |
| History of chemotherapy, yes | 239 (37.461) | 138 (32.547) | 0.103 |
| History of ACINV, yes | 228 (35.737) | 137 (32.311) | 0.072 |
| Type of interventional therapy, TACE | 335 (52.508) | 251 (59.198) | 0.135 |
| Interventional drug, platinum-based | 358 (56.113) | 233 (54.953) | 0.023 |
| History of intra-arterial interventional therapy, yes | 226 (35.423) | 133 (31.368) | 0.086 |
| Antiemetic regimen, single-agent | 217 (34.013) | 158 (37.264) | 0.068 |
| Anxiety, yes | 286 (44.828) | 189 (44.575) | 0.005 |
| Anticipatory nausea and vomiting, yes | 306 (47.962) | 189 (44.575) | 0.068 |
| Sleep duration, <7 h | 382 (59.875) | 235 (55.425) | 0.090 |
| History of motion sickness, yes | 138 (21.630) | 73 (17.217) | 0.110 |
| Existing comorbidity, yes | 281 (44.044) | 156 (36.792) | 0.147 |

Abbreviations: ACINV, acute chemotherapy-induced nausea and vomiting; BCLC, Barcelona Clinic Liver Cancer; SMD, standardized mean difference; TACE, transarterial chemoembolization.

For binary predictors, the SMD was calculated from the displayed category. For multi-category predictors, the reported value is the maximum absolute SMD across categories. An absolute SMD <0.100 is commonly interpreted as indicating negligible imbalance.

**Table S5. Stability of source-level predictor selection across the outer five-fold cross-validation**

| **Prespecified predictor** | **Selected in outer folds, *n*/5** | **Selection frequency** | **Retained in final full-training-set LASSO** |
| --- | --- | --- | --- |
| Sex | 5/5 | 100% | Yes |
| Age | 5/5 | 100% | Yes |
| Body mass index | 0/5 | 0% | No |
| Smoking history | 2/5 | 40% | Yes |
| Alcohol consumption | 0/5 | 0% | No |
| History of morning sickness | 0/5 | 0% | No |
| History of motion sickness | 0/5 | 0% | No |
| Existing comorbidity | 0/5 | 0% | No |
| Tumour stage (BCLC stage) | 1/5 | 20% | No |
| History of chemotherapy | 0/5 | 0% | No |
| History of ACINV | 5/5 | 100% | Yes |
| Type of IAIT | 5/5 | 100% | Yes |
| Interventional drug | 5/5 | 100% | Yes |
| History of IAIT | 5/5 | 100% | Yes |
| Antiemetic regimen | 5/5 | 100% | Yes |
| Anxiety | 5/5 | 100% | Yes |
| Anticipatory nausea and vomiting | 5/5 | 100% | Yes |
| Sleep duration | 5/5 | 100% | Yes |

Abbreviations: ACINV, acute chemotherapy-induced nausea and vomiting; BCLC, Barcelona Clinic Liver Cancer; IAIT, intra-arterial interventional therapy.

Note: Selection frequency was defined as the number of outer-loop training folds in which a predictor was retained by the fold-specific LASSO model at the penalty selected using strict inner five-fold cross-validation. Imputation, encoding and standardization were refitted within each LASSO training partition. For categorical predictors, a source-level predictor was considered selected when at least one encoded coefficient was non-zero. The final predictor set was determined by refitting the complete pipeline in the full training set using the one-standard-error rule (λ₁ₛₑ = 0.01869843). Outer-fold selection frequency describes stability and was not used as a voting rule for final predictor inclusion.

**Table S6. Pairwise comparison of AUC values among candidate ACINV models based on outer five-fold out-of-fold predictions in the training set.**

| ***P* values**  **Z values** | | **LR** | **DT** | **RF** | **SVM** | **XGBoost** | **KNN** | **NB** |
| --- | --- | --- | --- | --- | --- | --- | --- | --- |
|  |  | **0.865** | **0.893** | **0.941** | **0.931** | **0.933** | **0.913** | **0.853** |
| **LR** | **0.865** | NA | 0.051 | <0.001 | <0.001 | <0.001 | <0.001 | 0.043 |
| **DT** | **0.893** | 1.954 | NA | <0.001 | <0.001 | <0.001 | 0.103 | 0.007 |
| **RF** | **0.941** | 6.624 | 4.619 | NA | 0.0496 | 0.030 | <0.001 | <0.001 |
| **SVM** | **0.931** | 5.985 | 3.539 | −1.964 | NA | 0.789 | 0.010 | <0.001 |
| **XGBoost** | **0.933** | 6.057 | 4.058 | −2.167 | 0.267 | NA | 0.010 | <0.001 |
| **KNN** | **0.913** | 4.532 | 1.631 | −3.569 | −2.571 | −2.584 | NA | <0.001 |
| **NB** | **0.853** | −2.025 | −2.698 | −7.073 | −6.419 | −6.446 | −4.922 | NA |

Abbreviation: LR: logistic regression; DT: decision tree; RF: random forest; XGBoost: extreme gradient boosting; SVM: support vector machine; NB: naive bayes; KNN: k-nearest neighbor; AUC: area under receiver-operating curve; ACINV: acute chemotherapy-induced nausea and vomiting.

**Table S7. Confusion matrices derived from outer five-fold OOF predictions and held-out internal validation.**

| **Model/dataset** | **True negatives** | **False positives** | **False negatives** | **True positives** |
| --- | --- | --- | --- | --- |
| LR, training-set OOF | 295 | 117 | 30 | 196 |
| DT, training-set OOF | 340 | 72 | 29 | 197 |
| RF, training-set OOF | 353 | 59 | 21 | 205 |
| SVM, training-set OOF | 353 | 59 | 28 | 198 |
| XGBoost, training-set OOF | 357 | 55 | 30 | 196 |
| KNN, training-set OOF | 335 | 77 | 27 | 199 |
| NB, training-set OOF | 284 | 128 | 38 | 188 |
| Final RF, held-out internal-validation set | 233 | 37 | 9 | 145 |

Abbreviations: LR, logistic regression; DT, decision tree; RF, random forest; SVM, support vector machine; XGBoost, extreme gradient boosting; KNN, k-nearest neighbour; NB, naive Bayes; OOF, out-of-fold.
